# Investigating the relationship between personality traits of hardiness and perfectionism with stress and psychosomatic symptoms: a cross-sectional study among nurses in Iran

**DOI:** 10.1186/s40359-024-01832-4

**Published:** 2024-06-01

**Authors:** Mandana Abdolkarimi, Mohsen Sadeghi-Yarandi, Parisa Sakari

**Affiliations:** 1 Department of Clinical Psychology, Faculty of Medicine, Tonekabon Branch, Islamic Azad University, Tonekabon, Iran; 2 Department of Occupational Health, School of Public Health, Tehran University of Medical Sciences, Tehran, Iran

**Keywords:** Hardiness, Perfectionism, Stress, Psychosomatic Symptom, Nurses

## Abstract

**Background:**

Medical and health sector employees are always exposed to physical and psychological risk factors, which affects their personal, social and professional performance. It’s important to explores the intricate interplay between personality traits, stress levels, and psychosomatic symptoms among nurses as one of the most sensitive jobs in society. Therefore, the present study aimed to investigating the relationship between the personality traits of hardiness and perfectionism with stress and psychosomatic symptoms among nurses.

**Methods:**

This cross-sectional study was conducted among 340 nurses in Mazandaran, Iran in 2022–2023. The instruments utilized to collect data included four questionnaires, namely Cubasa Hardiness Questionnaire, Tehran Multidimensional Perfectionism Questionnaire, Nursing Stress Questionnaire and Takata and Sakata Psychosomatic Questionnaire. The structural equations modeling was used for path analysis. All analyzes were done using SPSS V.25.0 and AMOS V.24.0 software.

**Results:**

The results of the present study revealed that the prevalence of psychosomatic symptoms and stress in nurses was high, and all path coefficients were significant, except for the paths of commitment to stress, challenge to psychosomatic symptoms, self-orientation to psychosomatic symptoms, and community-orientation to psychosomatic symptoms. The results showed that in the final model, the highest coefficient (0.807) is assigned to the other-oriented perfectionism path to psychosomatic symptoms. The weakest coefficient (-0.276) is related to the path of the hardiness component of the challenge to stress. The current research examined the fitting of the proposed model and the suitability of the proposed model was confirmed.

**Conclusion:**

The results of the present study revealed that psychological factors such as personality traits of hardiness, and perfectionism are among the important and influencing parameters on occupational stress, and psychosomatic symptoms and as a result the efficiency and effectiveness of nurses in working environments. Therefore, it is absolutely necessary to implement mitigating and control measures to reduce the mentioned risk factors among nurses in medical settings.

## Background

Nurses exposed with many stressful factors in the work environment. Recent studies have shown that occupational stress not only disturbs the psycho-physical well-being of nurses and affects various aspects of their work life and personal life, but is also a risk parameter for patient safety and nursing quality [1, 2]. This group of community workers, daily face with many stressors in the workplace such as high physical and mental workload, personal conflicts, shift work, lack of suitable support, conflict with other medical staff in decision making, and conflict with patients’ companions. These factors can affect the various dimensions of nurses’ health [3–6]. Occupational stress has always been considered as an important psychological risk factor in the work environment [4]. Previous studies have shown that stress and psychosomatic symptoms are among the most important psychological risk factors that nurses suffer from due to the nature and sensitivity of their job duties [2, 7]. Previous research have reported that the prevalence of occupational stress among nurses is high, and a total of 88% of nurses are in medium and high stress levels, and this position can ultimately lead to an increase in the prevalence of psychosomatic symptoms among nurses [7]. Psychosomatic disorders are a wide group of diseases whose physical signs and symptoms are their main component. These problems and disorders refer to physical symptoms such as cardiovascular, respiratory, gastrointestinal, musculoskeletal, reproductive-urinary, skin disorders and other disorders such as migraine headaches, dizziness, excessive fatigue, and memory impairment. Problems in concentration include shortness of breath, nausea, vomiting, insomnia, etc., in which psychological events are closely related to physical symptoms [8, 9]. In addition, there are unknown mental or brain mechanisms that cause minor or detectable changes in neuro-chemistry, neuro-physiology, neuro-immunology, and cause the occurrence of these diseases [10].

Stress has always been considered as one of the most important risk factors in the emergence and formation of various physical and mental diseases and the death of people [4, 5, 11, 12]. Stress, anxiety and depression are among the most important psychological risk factors that can strongly affect different physical, cognitive and social dimensions of people in the work environment and family life [13, 14]. Stress is a force or pressure that can cause disruption and disintegration of the balance of the system or organism. Clinical findings show that mental stress caused by daily life events gradually affects the activity of different body systems and disrupts their function, or by weakening the immune system, it increases the susceptibility to mental and physical diseases. Common symptoms of stress are fatigue, headache, muscle tension, digestive disorders and dizziness [15]. Some studies supported the relationship between personality traits of the five big factors and physical health, and related stress [16, 17].

Moreover, perfectionism and hardiness are two personality traits that can be observed in the nursing community. Perfectionism means setting very high standards for performance and great effort to achieve them, which is associated with extreme critical self-evaluation and high sensitivity to mistakes [18]. The maladaptive dimension of perfectionism is related to perfectionistic evaluations and negative consequences such as anxiety and depression [19, 20]. Researchers state that perfectionism has three dimensions: self-oriented perfectionism, other-oriented perfectionism, and community-oriented perfectionism [21].

According to the definition provided by recent studies about hardiness; “Hardiness " is a collection and system of character traits that act as a source of resistance against stressful events in life [22]. Hardiness is known as the most important moderating source of the negative effects of stress, and has a positive effect on a person’s health [22, 23]. People who have lower hardiness will suffer from coronary heart disease, cholesterol and blood pressure in the long run [24]. A study found that training hardiness to nurses helps prevent burnout and stress [25].

Stress and psychosomatic symptoms are common among healthcare professionals, especially nurses who work in high-pressure environments. Several studies have explored the relationship between personality traits and stress among healthcare professionals, but little is known about the role of hardiness and perfectionism in this context. Due to the importance of the mentioned issue and the lack of comprehensive studies in this field, as well as the lack of studies based on the extraction of causal relationships between mentioned parameters, the present study aimed to investigating the relationship between the personality traits of hardiness and perfectionism with stress and psychosomatic symptoms among nurses in Mazandaran province, Iran.

## Subjects and methods

### Study design

This descriptive-analytical and cross-sectional study was conducted among 340 nurses in Mazandaran, Iran in 2022–2023 (from October 2022 to April 2023 continuously). The sample size was calculated according to the size of the statistical population using Cochran’s formula with an error level of 5%. This sample was randomly selected from all male and female nurses in Mazandaran province. In order to select the study sample, firstly, nurses from each department were selected by a stratified sampling, and then, using the simple random sampling method, the study subjects were selected from each department. During the current study, the required sample size of 310 people was determined and according to the prediction of 10% dropout rate, 360 people were selected to participate in the study. Finally, 340 nurse were evaluated in the present study (response rate: 94.4%). The participants were selected from a large hospital in Mazandaran province, Iran.

The inclusion criteria included being employed in the treatment and service sectors, at least two years of work experience, and the absence of psychological diseases. The presence or absence of mental illnesses of the study subjects was determined by examining the medical records of the employed persons. The exclusion criteria were the absence of sufficient consent to participate in the study. The participants completed the informed consent form before entering the study and were able to leave at any stage of the study in case of insufficient satisfaction. Before starting the study, participants were given the necessary training about the purpose of the study and familiarization with the questionnaires and how to complete them.

After determining the appropriate questionnaires, the questionnaires were designed electronically with the necessary explanations. After going through the procedures and obtaining permission from the relevant medical centers, the samples were randomly selected in medical centers and hospitals. The nurses who participated in this research belonged to most of the medical centers in terms of distribution, and this feature of the sample made it possible to generalize to the society better, thereby strengthening the external validity of this study. First, it was tried to gain participation, cooperation and trust, and the importance of the research was explained to them, and before sending the questionnaires, explanations were given about how to answer the questionnaires, and they were assured that the answers would remain confidential. The participants were asked to answer all the questions with patience and individually. Finally, the questionnaires registered in the relevant site were coded for analysis. The following questionnaires were used to check the studied components.

### Data collection tools

#### Kubasa hardiness questionnaire

Hardiness questionnaire was prepared and adjusted by Kobasa et al. in 1979 to measure hardiness from (Personal Viewpoints Survey Scale). This scale is a 50-item questionnaire that includes three subtests. It is also based on a Likert scale and has a range from 0 to 3 (not true at all, somewhat true, almost true, and completely true). This scale includes three components of commitment with 16 items, challenge with 17 items and control with 17 items.

If the scores of the questionnaire are between 0 and 66, it indicates low hardiness, scores between 67 and 132 indicate moderate hardiness, and scores 133 and above indicate high hardiness. The validity and reliability of this tool has been confirmed in previous studies. The reliability coefficient of control, commitment, and challenge components were 0.70, 0.52, and 0.52, respectively [26]. During the present study, Cronbach’s alpha values for three components of control, commitment, and challenge were obtained as 0.72, 0.70, and 0.79, respectively.

#### Tehran Multidimensional Perfectionism Scale (TMPS)

The Tehran Multidimensional Perfectionism Scale is a 30-question test in which 10 items measure self-oriented perfectionism, 10 items measure other-oriented perfectionism, and the last 10 items measure society-oriented perfectionism in a 5-point Likert scale (from 1 to 5) measures. The minimum and maximum score of the subject in the three sub-scales is 10 and 50, respectively; That is, the person who gets a score of 10 has the lowest level of perfectionism and the person who gets a score of 50 has the highest level of perfectionism in each of the three dimensions of perfectionism. The scaling method for all data is reversed. That is, the option “I completely agree” will be assigned a score of 5 and the option “I completely disagree” will be assigned a score of 1. The validity and reliability of this tool has been confirmed in previous studies.

Cronbach’s alpha obtained for self-oriented perfectionism was 0.90, for other-oriented perfectionism was 0.9, and for tolerance society perfectionism was 0.8, which shows the high internal consistency of the scale. The correlation coefficients for self-oriented perfectionism were 0.85, for other-oriented perfectionism 0.79 and for society-oriented perfectionism 0.84 were significant at *p* < 0.001 level, which is a sign of high retest reliability of the Iranian form of the scale [27]. During the present study, Cronbach’s alpha values for three components of self-oriented perfectionism, other-oriented perfectionism, and society-oriented perfectionism were obtained as 0.88, 0.90, and 0.82, respectively.

#### Nursing stress scale (NSS)

The Nursing Stress Questionnaire is the first tool that was created to measure nursing stress instead of general occupational stress. This questionnaire has 34 questions. The questions of this questionnaire are scored in seven range: suffering and death of the patient with 7 statements, conflict with doctors with 5 statements, insufficient preparation with 3 statements, lack of support resources with 3 statements, conflict with other nurses with 5 statements, work pressure with 6 statements, and treatment uncertainty with 5 statements. The retest reliability of this questionnaire was reported by Lee and et al. as 0.81 [28]. Pyne reports the Cronbach’s alpha coefficient of this questionnaire as 0.89 [29]. Lee and et al., report the reliability of the subscales of this questionnaire in a range between 0.67 and 0.79, and all the studies show the high reliability of this scale [28]. During the present study, Cronbach’s alpha values for this tool was obtained as 0.80.

#### Takata and Sakata psychosomatic questionnaire

The scale of psychosomatic complaints by Takata and Sakata consists of 30 questions and has a single-factor structure that was used to measure psychosomatic complaints. The scoring of the questionnaire is in the form of a 4-point Likert scale, where 0, 1, 2, and 3 points are considered for the options “never”, “rarely”, “sometimes” and “frequently”. The minimum possible score will be 0 and the maximum will be 90. A score between 0 and 30 indicates the amount of psychosomatic complaints is low. A score between 30 and 45 indicates the amount of psychosomatic complaints is average. A score higher than 45 indicates the amount of psychosomatic complaints is high.

The creators of this scale obtained its concurrent validity in two separate studies, 0.64 and 0.65, by calculating its correlation with the Goldberg mental health scale. Also, factor analysis was used to check the validity of the scale structure. The correlation between the parts of the scale was also reported as 0.50 or more in three different implementations by the creators of the scale [30]. During the present study, Cronbach’s alpha values for this tool was obtained as 0.73.

### Data analysis

In order to analyze the data, we first checked the descriptive statistics of the demographic variables and then checked the hypotheses with inferential statistics. Kolmogorov-Smirnov test was used to check the normal distribution of the data. In the inferential statistics section, the structural equations modeling in the form of path analysis has been used to examine the research model and hypotheses. On the basis of this model, the variables of personality traits of hardiness and perfectionism are considered as predictive and exogenous (independent) variables, and stress variables and psychosomatic symptoms are considered as endogenous (dependent) variables. All tests were checked at a significance level of 0.05. All analyzes were done using IBM SPSS Version 25.0 and AMOS Version 24.0 software.

## Results

### Descriptive statistics

The results of the present study demonstrated that 74.8% of participants were female and 25.2% were male. In terms of marital status, 39.5% were single, 50% were married, and 10.5% were divorced. 63.8% had no children, 22.9% had one child, and 13.3% had two children (Table 1). Also, 16.7% of subjects were under 25 years old, 57.1% were between 25 and 35 years old, 19.5% were between 35 and 45 years old, and 6.7% were between 45 and 55 years old.

**Table 1:** Frequency of child number groups

Number of children	Frequency	Frequency Percentage	Cumulative frequency
Without children	217	63.8%	63.8%
1	78	22.9%	86.7%
2	45	13.3%	100%
Total	340	100%	-

According to Table 2, 12.9% of nurses worked less than 20 h, and 23.3% worked more than 50 h in a week.

**Table 2:** Frequency of working hours’ groups

Working hours in a week	Frequency	Frequency Percentage	Cumulative frequency
Less than 20	44	12.9%	12.5%
20–30	23	6.7%	19.6%
30–40	128	37.6%	57.2%
40–50	66	19.5%	76.7%
More than 50	79	23.3%	100%
Total	340	100%	-

In Table 3 the descriptive information (mean and standard deviation) related to the personality traits variables of hardiness, perfectionism, stress and psychosomatic symptoms are reported.

According to the results of the correlation matrix in Table 4, there was a negative and significant relationship between personality traits of hardiness and stress and psychosomatic symptoms (*p*-value < 0.05).

**Table 3:** Descriptive indices (mean and standard deviation) of research variables among nurses

Variable	Mean	SD	Max-Min	Skewness	Kurtosis
**Commitment** ^*^	29.09	12.34	48 − 10	-0.089	-1.143
**Challenge** ^*^	27.85	12.07	51 − 10	0.174	-1.123
**Control** ^*^	29.28	12.71	50 − 13	0.296	-1.342
**Self-Oriented** ^**^	31.17	9.94	47 − 15	0.115	-1.229
**Other- Oriented** ^**^	32.15	11.22	50 − 17	0.332	-1.301
**Society-Oriented** ^**^	32.56	11.15	49 − 15	0.124	-1.235
**Stress**	54.88	24.89	100 − 25	0.436	-1.211
**Psychosomatic**	50.78	24.57	90 − 16	0.280	-1.373

^*^ Personality traits of hardiness dimensions

^**^ Perfectionism dimensions

Also, there was a positive and significant relationship between the characteristics of perfectionism and stress and psychosomatic symptoms (*p*-value < 0.05). Correlation matrix between predictor and dependent variables of the final model are presented in Table 4.

**Table 4:** Correlation matrix between predictor and dependent variables of the final model

	1	2	3	4	5	6	7	8
1. Commitment	1							
2. Challenge	0.643^*^	1						
3. Control	0.509^*^	0.204^*^	1					
4. Self-oriented	-0.513^*^	-0.850^*^	-0.214^*^	1				
5. Other-oriented	-0.658^*^	-0.227^*^	-0898^*^	0.294^*^	1			
6. Society-oriented	-0.715^*^	-0.844^*^	-0.121^*^	0.828^*^	0.301^*^	1		
7. Stress	-0.454^*^	-0.379^*^	-0.662^*^	0.446^*^	0.563^*^	0.377^*^	1	
8. Psychosomatic	-0.863^*^	-0.577^*^	-0.549^*^	0.538^*^	0.757^*^	o.670^*^	0.295^*^	1

^*^p-value < 0.01

The value of Cronbach’s alpha of all research questionnaires was above 0.7, so the questionnaires have a high level of reliability and internal consistency to measure their indicators.

### Analytical statistics

To check the absence of multivariate outlier data, Mahalanobis d^2^ index was examined and significance levels less than 0.05 indicate the remoteness of the desired outlier data. Based on this index, there was no outlier data. To check the normality of several variables, Mardia’s normalized multivariate kurtosis value was used in the current research, this number was equal to 4.186, which is less than 80. The mentioned number was calculated through the formula p (*p* + 2). In this formula, p is equal to the number of observed variables, which was eight in this research [31].

Before examining the structural coefficients, the suitability of the proposed model was examined. The goodness-of-fit of the proposed model was evaluated based on the introduced fit indices. Considering that CMIN/DF values was smaller than 5 and RMSEA was less than 0.1, the goodness-of-fit of the proposed model was approved [32].

**Table 5:** Goodness-of-fit indices of the proposed and final model of the current research

Model fit indices	Acceptable range	First Model	Final Model
X^2^	-	3.055	8.649
df	-	1	4
p-value	< 0.05	< 0.001	< 0.001
CMIN/df	Good > 3, Acceptable > 5	3.055	2.162
RMSEA	< 0.08	0.099	0.074
PNFI	> 0.5	0.534	0.641
CFI	> 0.9	0.890	0.988
PCFI	> 0.5	0.534	0.641
IFI	> 0.9	0.896	0.988
GFI	> 0.9	0.874	0.970

Note: CMIN/DF: Chi-square/degree-of-freedom ratio; RMSEA: Root Mean Square Error of Approximation; PCFI: Parsimonious Comparative Fit Index; GFI: Goodness of Fit Index; PNFI: Parsimonious Normed Fit Index; IFI: Incremental Fit Index; CFI: Comparative Fit Index

Next, in order to improve the model, in the first step, non-meaningful paths (“commitment to stress, challenge to psychosomatic symptoms, self-orientation to psychosomatic symptoms and society-orientation to psychosomatic symptoms”) were removed. In the final step, by drawing the correlation between the covariance errors, the final model of the research was drawn. The results revealed that after the modifications, the final obtained model has a good fit. The goodness-of-fit indices are presented in Table 5.

**Fig. 1:**
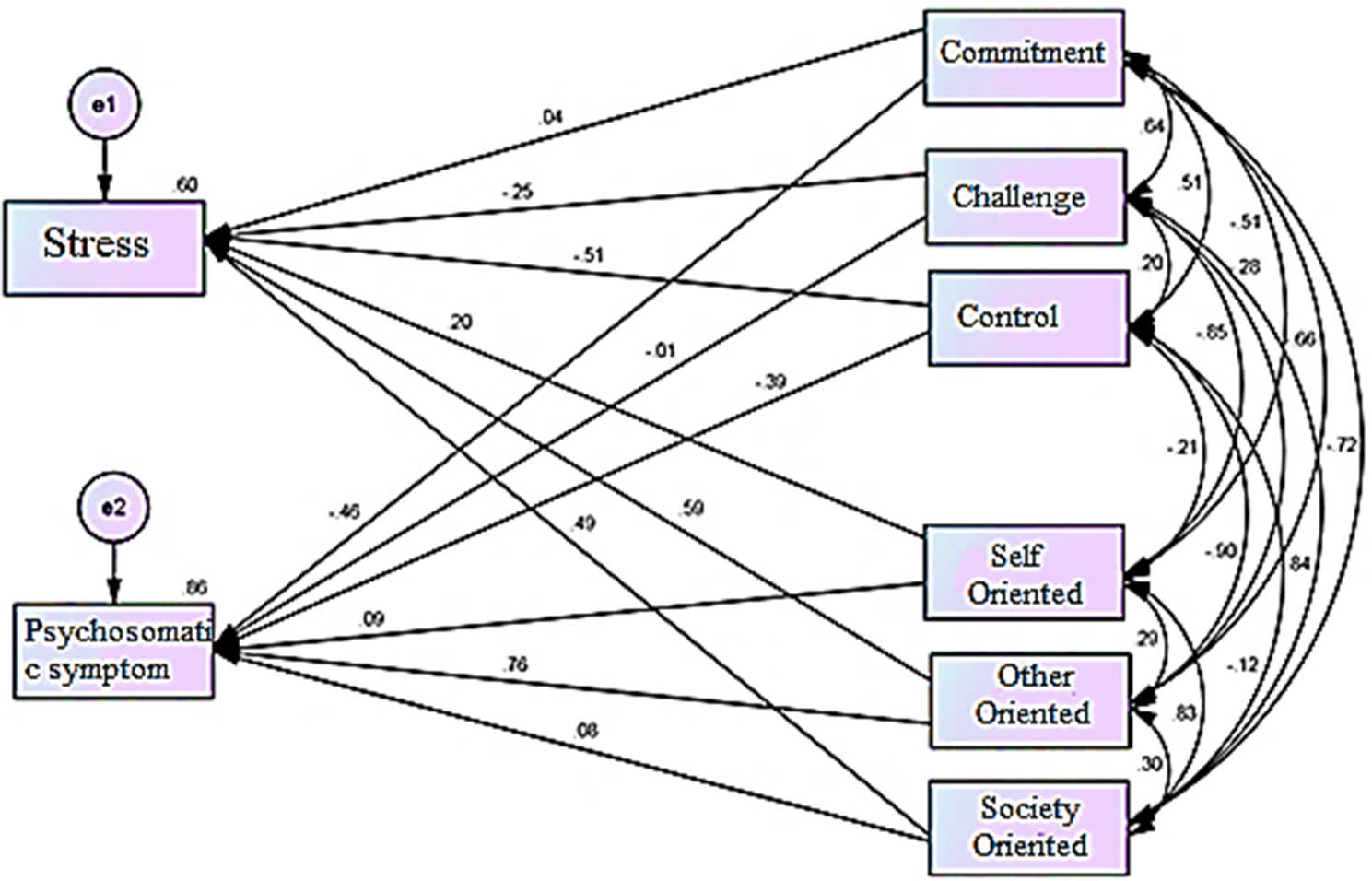
The standard coefficients of the proposed model of the structural relationship between the personality traits of hardiness and perfectionism with stress and psychosomatic symptoms in nurses

**Fig. 2:**
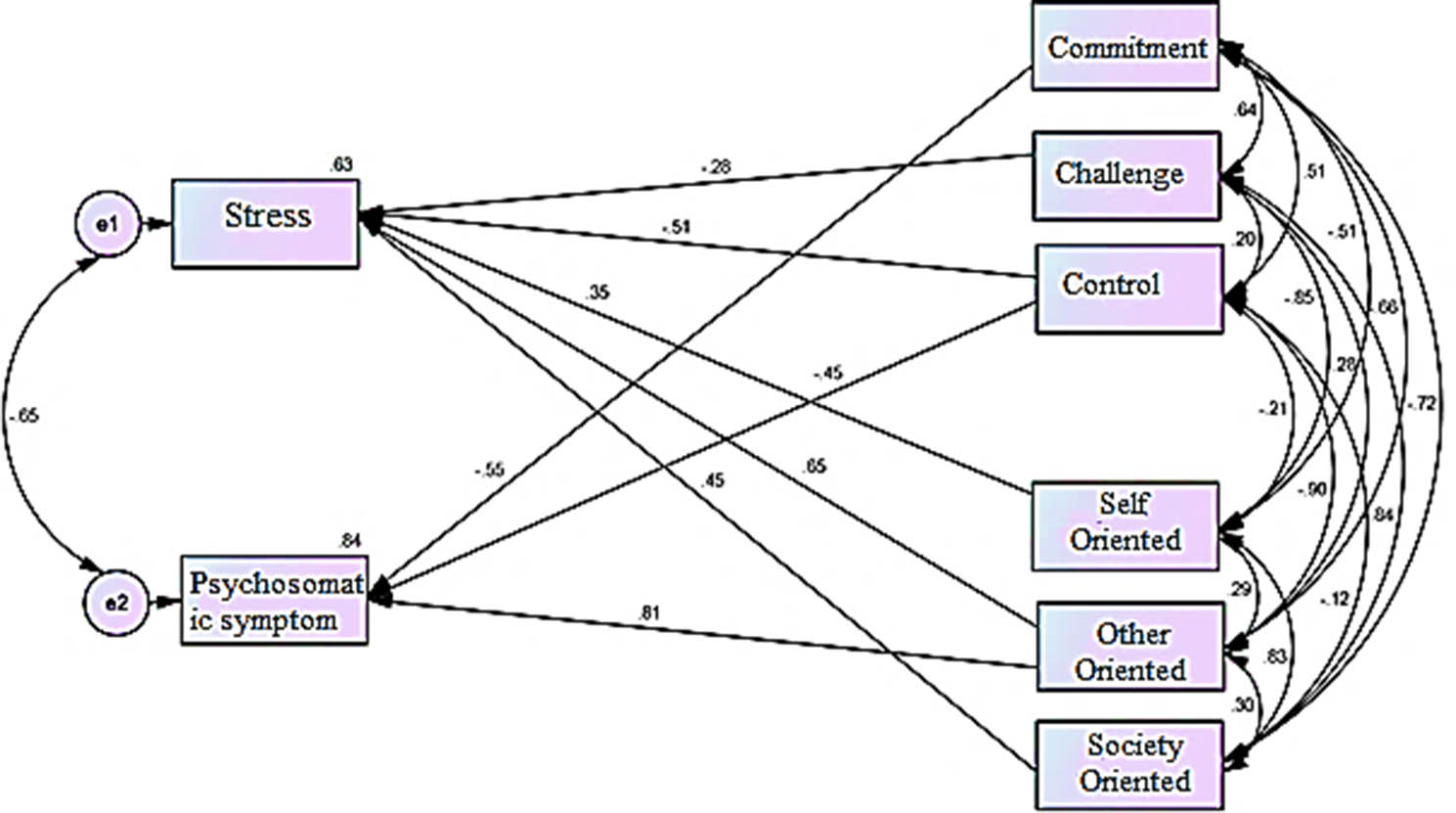
Standardized coefficients of the final (modified) model of the structural relationship between personality traits of hardiness and perfectionism with stress and psychosomatic symptoms in nurses

In Fig. 1, the initial model is drawn according to the existing hypothesis. In Fig. 2, the initial model is standardized and presented as the final model. According to Fig. 2, the coefficient of determination of stress variable and psychosomatic symptoms were equal to 0.630 and 0.840, respectively, which shows that the variables of personality traits of hardiness and perfectionism can predict 63% of stress changes and 84% of psychosomatic symptom changes, respectively, which was a strong rate. Therefore, according to the estimated indicators, it shows that the structural relationship of personality traits of hardiness and perfectionism with stress and psychosomatic symptoms in nurses was suitable.

In Figs. 1 and 2, the numbers on the paths are path weights or beta coefficients. In the final model, the highest coefficient (0.807) was assigned to the other-orbital path to psychosomatic symptoms, and the weakest coefficient (-0.276) was related to the path of challenge to stress.

The results of the direct relationships of the research variables in the proposed model show that in the whole sample, all path coefficients except for the paths of commitment to stress, challenge to psychosomatic symptoms, self-orientation to psychosomatic symptoms and community orientation to psychosomatic symptoms were statistically significant.

## Discussion

In this study, the path analysis model predicting stress and psychosomatic symptoms of the investigated nurses was drawn. On the basis of this model, the variables of personality traits of hardiness and perfectionism are considered as predictive and exogenous (independent) variables, and stress and psychosomatic symptoms are considered as endogenous (dependent) variables. Personality traits play a crucial role in shaping individuals’ responses to stress and their susceptibility to psychosomatic symptoms, especially in high-stress environments such as healthcare settings. Nurses with high levels of hardiness may exhibit greater coping mechanisms and adaptability in the face of work-related stressors, potentially reducing the likelihood of experiencing psychosomatic symptoms. Conversely, perfectionism, while often associated with high achievement and attention to detail, can also contribute to heightened levels of stress and maladaptive coping strategies in demanding professions like nursing. Thus, understanding the intricate interplay between hardiness, perfectionism, stress, and psychosomatic symptoms is essential for developing targeted interventions to support the mental well-being of nurses.

In the present study, there was a negative and significant relationship between personality traits of hardiness and stress and psychosomatic symptoms (Table 4). Petzold et al. also showed that psychological stress, anxiety, hardiness, and self-efficacy affect their quality of life and performance during the period when people get sick or are afraid of getting sick [33]. Also, the results of the present study were largely consistent with the studies of Bedo et al. [34].

Huang also concluded in a study that people with high hardiness have better health and perceive life changes in a more positive and challenging way than people who do not have this characteristic. In stressful situations, those with higher hardiness have better mental health than those with lower hardiness [35]. Hardiness is a personality trait characterized by a sense of commitment, control, and challenge. Nurses high in hardiness are more likely to perceive stressful situations as challenges to be overcome rather than threats. High hardiness can also enhance nurses’ resilience and coping abilities, allowing them to adapt more effectively to stressful situations and reduce the likelihood of experiencing psychosomatic symptoms.

In a part of the present study, contrary to the mentioned studies, the commitment component does not have a significant negative relationship with stress. For this reason, the high commitment factor, in other words, being committed does not reduce stress. While commitment to one’s job can be a protective factor against job stress in some cases, it is important to recognize that the relationship between commitment and job stress is complex and multifaceted. Individual differences, coping mechanisms, organizational support, job satisfaction, role clarity, and autonomy all play a role in determining how nurses experience and respond to job stress which can be also different in different societies.

Previous study revealed that hard people use problem-oriented coping methods and social support. When faced with problems, these people make a more accurate assessment of them and use problem-oriented strategies to solve problems. In fact, hardiness increases one’s personal efficiency in dealing with problems [34].

According to the results obtained, the present study is along the results of the study Basharat et al. [36] which showed that there is a positive relationship between other-oriented and community-oriented perfectionism with symptoms of depression and anxiety, and this finding is also consistent with the results of Stoeber et al. [37]; Smith et al. [38]; Cha et al. [39]; Chang et al. [40]; Sherry et al. [41]; Lasota and Kearney [42]; Lessin and Pardo [43]. Moreover, previous study mentioned that interpersonal relational role systems are an important support in reducing the relational stress experienced by nurses [44].

Perfectionism is a personality trait characterized by high standards, self-criticism, and concerns about mistakes. Nurses with perfectionistic tendencies may set unrealistic expectations for themselves and experience heightened levels of stress when they perceive they are falling short. Dimensions of perfectionism, such as self-oriented perfectionism (setting high standards for oneself), other-oriented perfectionism (expecting high standards from others), and socially prescribed perfectionism (believing others expect perfection), can influence how nurses experience job stress and psychosomatic symptoms. Other-oriented and socially prescribed perfectionism may contribute to interpersonal conflicts, feelings of inadequacy, and a sense of being overwhelmed, all of which can exacerbate job stress and psychosomatic symptoms.

Perfectionist people need to control their environment to prevent possible dangers. They think that mistakes reduce their control, so everything they do must be perfect. When this perfection does not exist, the worry about mistakes becomes more intense and it leads to an increase in stress, which in turn reduces efficiency and increases the possibility of new mistakes. The opinion of others is very important for perfectionist people (Society-Oriented- perfectionism).

Based on the listed results, the characteristics of other-oriented and community-oriented have a positive and significant effect on stress. However, no significant effect was observed between self-oriented perfectionism and stress. This shows that among the studied nurses, the opinion of the society and others about their performance is more important and causes most of them to experience job stress.

Therefore, the other-oriented and community-oriented perfectionism have a positive and significant effect on the stress of nurses. This result is in accordance with Terpanier et al. [45].

Regarding the relationship between the components of perfectionism and psychosomatic symptoms, other-oriented perfectionism has a positive and significant effect on psychosomatic symptoms. However, no significant effect was observed between self-oriented perfectionism and community-oriented characteristics with psychosomatic symptoms. According to the previous research studies, there was a significant correlation between perfectionism and migraine headache symptoms in the studied nurses in such a way that 30% of the variance of migraine headache symptoms could be explained by the dimensions of perfectionism. This finding is also consistent with the findings of the present research [46]. Also, the results of Abdollahi et al.‘s study among nurses in Tehran showed the significant relationship between self-compassion and perceived stress in nurses, which in line with the results of the present study [47].

This study can shed light on the complex dynamics between personality attributes, occupational stress, and psychosomatic manifestations within the specific context of nurses in Iran. The findings of this research offer valuable insights into the nuanced connections between personality traits like hardiness and perfectionism and their impact on stress levels and psychosomatic symptoms among healthcare professionals in Iran.

The results of this study may have significant implications for healthcare organizations and policymakers in such working environment, highlighting the need for targeted interventions that address the psychological well-being of nurses. By recognizing the role of personality traits in shaping responses to stress and contributing to psychosomatic symptoms, healthcare providers can tailor support programs and resources to enhance coping strategies and promote mental wellness among nursing staff. Moreover, the study underscores the importance of fostering a holistic approach to employee well-being that considers not only the external stressors in the work environment but also the internal psychological factors that influence how individuals perceive and respond to these stressors.

Finally, the following control measures can lead to reducing and adjusting the levels of occupational stress and psychosomatic symptoms among the nursing community:

1. Implement stress management programs: Hospitals and healthcare facilities can provide stress management programs specifically designed for nurses. These programs can include mindfulness training, relaxation techniques, and coping strategies to help nurses manage their stress levels effectively by adjusting their personality traits of hardiness and perfectionism level.
2. Increase staffing levels: One of the main reasons for stress among nurses is high workloads and understaffing. By increasing staffing levels, nurses can have more time to care for patients, reducing their stress levels and the likelihood of experiencing psychosomatic symptoms.
3. Provide mental health support: Hospitals should offer mental health support services for nurses, including access to counseling and therapy. This can help nurses address any underlying mental health issues contributing to their stress and psychosomatic symptoms.
4. Encourage work-life balance: Employers can promote work-life balance by implementing flexible scheduling options, providing paid time off, and encouraging nurses to take breaks during their shifts. Balancing work with personal time can help reduce stress and prevent burnout.
5. Offer training on coping mechanisms: Hospitals can provide training on coping mechanisms and resilience-building techniques for nurses to help them better manage stress and prevent psychosomatic symptoms. This training can include education on healthy lifestyle habits, self-care practices, and effective communication strategies.
6. Foster a supportive work environment: Creating a supportive work environment where nurses feel valued, appreciated, and respected can help reduce stress levels and improve overall well-being. Encouraging teamwork, open communication, and peer support can contribute to a positive workplace culture that promotes mental health and reduces psychosomatic symptoms.
7. Address organizational factors: Hospitals should also address organizational factors that contribute to stress among nurses, such as excessive paperwork, long working hours, and lack of resources. By addressing these issues, hospitals can create a more conducive work environment that supports nurses’ mental health and well-being.
8. Special counseling programs in the field of perfectionism and personality traits of hardiness: Despite the implementation of the above measures, occupational stress and subsequent psychosomatic symptoms among nurses are still unavoidable. Therefore, holding training workshops and individual counseling sessions for nurses on determining the levels of perfectionism, personality traits of hardiness, as well as determining the personality characteristics of people can be an effective step in predicting and controlling occupational stress levels and psychosomatic symptoms among nurses.

### Strengths and limitations of the study

During the present study, for the first time, the relationship between the personality traits of hardiness and perfectionism with stress and psychosomatic symptoms among nurses in Iran were investigated. The results of the present study can create a novel scientific insight in the field of risk factors affecting the prevalence of occupational stress and psychosomatic symptoms among nurses. Ultimately, by delving into the intricate interplay between personality traits, stress, and psychosomatic symptoms among nurses in Iran, this study paves the way for a more comprehensive understanding of the multifaceted challenges faced by healthcare professionals in demanding work settings.

Among the limitations of the present study can be the use of self-report tools in determining the values of the investigated parameters, the impossibility of investigating other predictors of job stress and psychosomatic symptoms such as job satisfaction, job burnout, mental workload, anxiety and other psychological and psycho-social risk factors due to time limitations, study of a limited number of nurses and the impossibility of studying other jobs in the medical staff, as well as the impossibility of conducting an interventional study due to time and economic limitations. Therefore, it is suggested that in the future, while considering confounding variables, researchers study the interaction of other psychological and psychosocial risk factors affecting the prevalence of occupational stress and psychosomatic symptoms in medical settings and, conduct interventional studies based on the obtained results. In addition, it is suggested that researchers implement control and mitigation measures and report the effectiveness of control measures. Also, due to the fact that the current study is cross-sectional, it was not possible to investigate the exact mutual cause and effect relationships between the parameters.

## Conclusion

The results of the present study revealed that psychological factors such as personality traits of hardiness, and perfectionism are among the important and influencing parameters on occupational stress, and psychosomatic symptoms and as a result the efficiency and effectiveness of nurses in working environments. Therefore, it is absolutely necessary to implement control measures to reduce the mentioned risk factors among nurses. Policymakers can consider incorporating these findings into broader efforts to improve the quality of healthcare work environments and reduce burnout among healthcare professionals.

## Data Availability

All data generated or analyzed during this study are included in this article. Further enquiries can be directed to the corresponding author.
